# Blood Pressure, Serum Glucose, Cholesterol, and Triglycerides in Dogs with Different Body Scores

**DOI:** 10.1155/2016/8675283

**Published:** 2016-12-12

**Authors:** Mauro José Lahm Cardoso, Rafael Fagnani, Carolina Zaghi Cavalcante, Marcelo de Souza Zanutto, Ademir Zacarias Júnior, Luciane Holsback da Silveira Fertonani, Jéssica Ragazzi Calesso, Maíra Melussi, Helena Pinheiro Costa, Eduardo Yudi Hashizume

**Affiliations:** ^1^Department of Veterinary Clinics, State University of Londrina, Londrina, PR, Brazil; ^2^Northern Paraná State University, Londrina, PR, Brazil; ^3^Pontifical Catholic University of Paraná, Curitiba, PR, Brazil; ^4^Northern Paraná University, Bandeirantes, PR, Brazil; ^5^Veterinary Space Life, Londrina, PR, Brazil

## Abstract

The objective of this research was to determine the frequency for the occurrence of MS in dogs, using the criteria determined, and to correlate the criteria of dogs that would characterize the MS with different body condition score (BCS). 271 dogs with different body scores were studied, with 101 dogs with BCS 4-5; 101 dogs with BCS 6-7; and 69 dogs with BCS 8-9. Among the dogs studied, 62 (22,87%) had two or more inclusion criteria for MS. Of these, 28 had BCS 6-7, while 34 dogs had BCS 8-9. Therefore, 27,72% of overweight dogs had inclusion criteria for MS and 49,27% of obese ones had two or more inclusion criteria for MS. When only overweight and obese dogs were considered as a total population, it was observed that 36,47% got inclusion criteria for the MS. No dog with BCS 4-5 showed two or more inclusion criteria for MS. The metabolic syndrome, according to the parameters for inclusion defined in the literature, was observed in 22,87% of the animals studied and in 36% of dogs overweight or obese. Furthermore, MS was most common in obese (49%) compared to overweight dogs (27%).

## 1. Introduction

Metabolic syndrome (MS) gained attention in human medicine because of the association with the development of* diabetes mellitus* and cardiovascular disease [1]. To characterize this MS is necessary for the presence of visceral (central) obesity in conjunction with dyslipidemia including the increase of triglyceride and decrease of HDL-cholesterol, hypertension, and glucose intolerance. In veterinary medicine MS in equines is well described and known to be a risk factor for the development of laminitis and other diseases [2]. Obese dogs may develop some components of the MS, including insulin resistance, hyperlipidemia, and mild hypertension, which improve with weight loss [3–5].

The consensus statement that defines the inclusion of the human patient as having MS [6] includes the presence of at least three to five of the following: triglycerides >150 mg/dL (>1,7 mmol/L); HDL-cholesterol <40 mg/dL (<1,3 mmol/L) in men or 50 mg/dL (<1,29 mmol/L) in women; arterial systolic pressure >135/85 mmHg; plasma glucose >100 mg/dL (5,6 mmol/L); increased waist circumference [6]. The first criteria suggested for the classification of MS in naturally obese dogs were BCS 7–9/9 points and two of the following of these criteria: triglycerides >200 mg/dL (2,3 mmol/L); total cholesterol >300 mg/dL (7,8 mmol/L); systolic blood pressure >160 mmHg; plasma glucose >100 mg/dL (5,6 mmol/L); previous diagnosis of* diabetes mellitus* type 2 [7].

The objective of this research was to determine the frequency for the occurrence of MS in dogs, using the criteria determined by Tvarijonaviciute et al. [7], and to correlate the criteria of dogs that would characterize the MS with different BCS.

## 2. Material and Methods

Between January 2013 and October 2014, 271 healthy dogs attended in Veterinary Hospitals of the Universidade Estadual do Norte do Paraná (Bandeirantes, Paraná State) and Universidade Estadual de Londrina (Londrina, Paraná State) and the Espaço Vida Veterinária (Londrina, Paraná State) were selected, to participate in this research. All owners signed an informed consent to participate in this research. This study was approved by ethics board of the animal experiments, State University of Northern Paraná, School of Veterinary Medicine, Department of Production and Veterinary Medicine, Bandeirantes, Paraná, Brazil, and is in accordance with the ethical principles of animal experimentation.

The animals remained fasting for 12 hours prior to the collection of blood samples. The dogs were weighed and had their body condition score (BCS) determined using the system of 9 points proposed by Laflamme [8] and distributed in these groups: control group (BCS 4-5/9), overweight group (BCS 6-7/9), and obese group (BCS 8-9/9). An initial screening to evaluate the overall health was performed including history, physical exam, blood count, serum chemistry profile, total thyroxin (TT4), free thyroxin (FT4), thyroid stimulating hormone (TSH), and suppression test low dose of dexamethasone (performed only in dogs over or equal to the BCS 6).

The animals with endocrine, liver, kidney and/or heart disease, heart failure congestive, and pancreatitis and those receiving glucocorticoids systemically or topically, anticonvulsant, and hypotensive drugs were excluded from the study. To exclude dogs with hypothyroidism FT4, TT4, and TSH tests were performed. Dogs that showed a decrease in TT4 concentration were excluded, even with no increase in TSH. To exclude dogs with Cushing's disease suppression test was performed with low dose of dexamethasone. The blood count, urinalysis, serum chemistry profile, and abdominal ultrasonography were used to rule out kidneys, liver, and pancreas diseases. The electrocardiography, echocardiography, and thoracic radiography were used to rule out heart diseases.

Definition of metabolic syndrome is as follows: (1) body score 7–9/9 points and two of the following criteria: (2) triglycerides >200 mg/dL (2,3 mmol/L); (3) total cholesterol >300 mg/dL (7,8 mmol/L); (4) systolic blood pressure >160 mmHg; (5) plasma glucose >100 mg/dL (5,6 mmol/L); (6) previous diagnosis of* diabetes mellitus* type 2.

### 2.1. Systolic Blood Pressure

Systolic blood pressure (SBP) was obtained by noninvasive method using Doppler flowmeter (Doppler Vascular DV 610, Medmega Industry Medical Equipment) as described previously [9]. SBP in five different measurements was obtained and the average was calculated, discarding the lowest and the highest. All dogs were in sternal recumbency and cuffs were selected according to the right forelimb diameter (40% of the circumference of the limb).

### 2.2. Sample Collection

Blood samples were collected by jugular venipuncture after 12 hours fasting in tubes containing clot activator and gel bottles containing sodium fluoride. The samples were centrifuged at 2000 G/minute for 10 minutes up to one hour after collection. After obtaining serum and plasma, the samples were stored in a freezer at −70°C. Serum cholesterol, triglycerides, and plasma concentrations of glucose were performed on automated chemistry analyzer (Catalyst One Chemistry Analyzer, IDEXX Laboratories, USA) using commercial kits and using the methodology recommended by the manufacturer, with a coefficient of variation within and between-run <2% for all analyses. Serum cortisol measurements (Total T4 RIA 125 kit, Siemens Healthcare Diagnostics, Tarrytown, NY), FT4 (Free T4, Two-Step, 125 I RIA Kit, DiaSorin, Stillwater, MN), and TSH (Canine TSH IRMA; Diagnostic Products Corp., Los Angeles, CA) were performed according to manufacturer's recommendations.

### 2.3. Statistical Analysis

The retrospective study evaluated a population of 6,729 dogs, where 2,557 were overweight and obese, and of these 271 (10.6%) met the inclusion and exclusion criteria.

The animals were classified into four groups: three according to the BCS: BCS 4-5 (ideal weight or control group), BCS 6-7 (overweight group), and BCS 8-9 (obese group); and the fourth group were the dogs with two or more inclusion criteria for MS. The inclusion of animals in the fourth group did not exclude groups based on BCS. The experimental design was completely randomized and considered any differences on serum levels of cholesterol, triglycerides, glucose, blood pressure, and age among the four groups. Variables not normal and equality by the Kolmogorov-Smirnov test and Liliefors test (*p* < 0,05). Thus, the differences between groups were assessed by Kruskal-Wallis test (*p* < 0,05). The percentage of dogs with hypercholesterolemia (>300 mg/dL), hypertriglyceridemia (>200 mg/dL), hyperglycemia (>100 mg/dL), and pressure (>160 mg/hg) was compared among the four groups tested by Chi-square test (*p* < 0,05). The occurrence of neutered dogs and ratio male/female was also compared among the four groups by Chi-square test (*p* < 0,05). The likelihood of these events occurring among the four groups was calculated using the odds ratio of the formula: (*p*/(1 − *p*))/(*q*/(1 − *q*)), where *p* is the probability that an event occurs; *q* is the probability that the second event occurs. Within the group of dogs with two or more MS criteria the BCS with serum levels of cholesterol, triglycerides, glucose, and blood pressure were correlated by nonlinear Spearman correlation.

## 3. Results

The dogs were 2–14 years old, with 95 between 2 and 6 years old, 127 between 6 and 10 years old, and 49 between 10 and 14 years old. Crossbred dogs (102) were included as well as Beagle (6), Border Collie (8), Boxer (9), Chow-Chow (1), Cocker Spaniel (8), Fila Brasileiro (1), Golden Retriever (3), Labrador Retriever (12), Lhasa Apso (16), Maltese (2), English Mastiff (1), German Shepherd (1), Pekingese (1), American Pit Bull Terrier (16), Poodle (23), Pug (3), Rottweiler (7), Schnauzer (8), Shih-Tzu (7), German Spitz (7), Dachshund (16), Brazilian Terrier (1), and Yorkshire Terrier (12).

**Figure 1:**
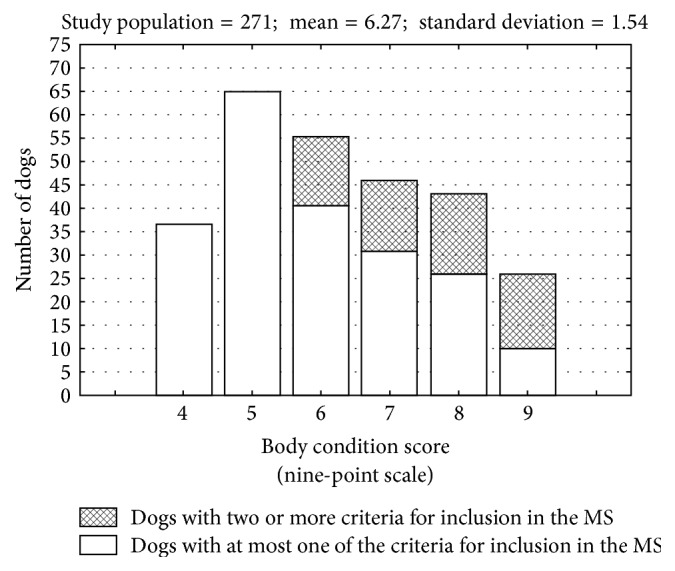
Absolute frequency of 271 dogs grouped according to the BCS and separated according to the number of criteria used for inclusion in the metabolic syndrome (MS).

Of the 271 dogs studied 62 (22,87%) had two or more MS inclusion criteria. Of these, 28 had BCS 6-7, while 34 dogs had BCS 8-9. Therefore, 27,72% of overweight dogs had MS inclusion criteria and 49,27% of obese had two or more MS inclusion criteria (Figure 1). This difference in proportion was considered significant in Chi-square test. No dog with BCS 4-5 showed two or more MS inclusion criteria.

Table 1 shows the distribution of cholesterol, blood glucose, triglycerides levels, and the BCS. For dogs in the group with two or more inclusion criteria for MS, fasting blood glucose was above 100 mg/dL in 39 (62,90%), hypertriglyceridemia in 57 (91,94%), hypercholesterolemia in 51 (82,26%), and systolic hypertension (SH) in 24 (38,71%). In these animals, 19 had higher triglycerides 400 mg/dL (moderate hypertriglyceridemia) and only two dogs had values greater than 1000 mg/dL (severe hypertriglyceridemia). In 20 dogs serum cholesterol concentration greater than 500 mg/dL (moderate hypercholesterolemia) was detected and eight dogs had values above 750 mg/dL (severe hypercholesterolemia). Of the 62 dogs in the MS group, 14 had moderate increase in the systolic hypertension (160–179 mmHg) and 10 had severe increase (>180 mmHg).

The breed distribution of the 62 dogs with MS was American Pit Bull Terrier (4/16), Beagle (4/6), Boxer (1/9), Cocker Spaniel (2/8), Labrador (2/12) Lhasa Apso (3/16), Maltese (1/2), Poodle (6/23), Pug (2/3), Rottweiler (1/7), Schnauzer (5/8), Shih-Tzu (2/7), Spitz German (1/7), Dachshund (1/16), Brazilian Terrier (1/1), Yorkshire Terrier (7/12), and crossbreed dogs (19/102).

The majority correlations occurring in the 62 dogs with MS were weak (Table 2), and only moderate association between serum concentrations of glucose and triglyceride (0,39), cholesterol and age (12 : 31), and BCS and SBP (0,38) occurred. The correlation between these variables is represented by polynomials expressed in Tables 2 and 3.

Table 3 shows that the frequency of hyperglycemia in dogs with BCS 4-5 was lower than other groups. The probability of a dog with MS to present hyperglycemia (37,09%) is the same as compared with BCS 6-7 and BCS 8-9. With regard to dogs with BCS 4-5 the ratio was statistically different and decreased to 14,85% the likelihood of a dog with these scores developing hyperglycemia. The chance of dogs with BCS >5 or MS present hyperglycemia was on average 2,91 times higher when compared to BCS 4-5.

Dogs with MS are 17,9% more likely to develop hypercholesterolemia than other groups (increased from 29,52% to 79,03%). In this group, the chance of animals having hypertriglyceridemia increases by 28,04 times compared to the group BCS 4-5.

The frequency of hypertension was higher in groups with BCS 8-9 and MS with values of 27,54% and 38,71%, respectively, and without difference when compared. But when compared to dogs of other groups there is statistical difference (*p* < 0,05). However, when comparing BCS 8-9 and MS group with others, the chance of developing hypertension decreases by 8,33 times (odds ratio) in BCS 4-5 and 6-7 group.

Neutering was associated with the BCS and the dogs with MS. In the MS group the probability of a dog being neutered was 79,03%, presenting 4,09 more chances (odds ratio) of having neutered dogs with MS than neutered dogs with BCS 4-5 and BCS 6-7. The proportion of females and males did not differ between groups according to the BCS and MS inclusion criteria.

## 4. Discussion

101 dogs (37,27%) had ideal weight, 101 (37,27%) were overweight, and 69 (25,46%) were obese and therefore 62,73% of the studied population was overweight, with results higher than previous studies [10, 11]. Nevertheless, the results do not reveal the prevalence of obesity in all three veterinary centers where the survey was conducted. In these places, the frequency of dogs overweight and obese was 47% in unpublished data.

The inclusion criteria in the MS group were found in Pugs (66%), Schnauzers (62,5%), Beagles (66%), Labradors (17%), and Yorkshire Terriers (58%) overweight and obese, admittedly breeds with higher risk for obesity [10, 11]. However, due to the small number of dogs of each breed in MS group multivariate linear regression could not be performed to assess whether it was adiposity, breed, or metabolic factors that influenced the development of the changes in the MS. The dogs that met the criteria for metabolic syndrome were of different races and sizes, and 28 of 62 dogs (45%) weighed even 10 kg of live weight. The findings suggest that small dogs have higher adiposity than medium or large dogs. However, more than 50% of the study population consisted of small dogs.

Elevation of cholesterol and triglycerides with the increases in the BCS can indicate that the degree of fat has influence on these substances, though the median values of cholesterol and triglycerides of MS group have slight increase. Serum triglyceride concentrations in dogs with BCS 8-9 and dogs with MS are similar in other studies [7, 12, 13] and higher in others [14, 15]. The median values of cholesterol in the BCS 8-9 group were similar to described previously [14, 15] but in the MS group were higher when compared to these same studies. Pancreatitis, hepatobiliary disease, atherosclerosis, eye damage [12], insulin resistance [16], and seizures are complications associated with hyperlipidemia, but none of the animals had these complications. In dogs overweight and obese 27 with triglycerides above 445 mg/dL and 19 (70,37%) dogs from MS group were observed. According to Verkest et al. [5], plasma concentrations above 445 mg/dL have been associated with increased immunoreactive lipase activity in the risk of developing pancreatitis.

The glucose values were higher (*p* < 0,05) in the MS group, probably due to insulin resistance. In dogs with BCS 6-7/8-9 and 9/9 median glucose was below 100 mg/dL, and similar results were described in which the average blood glucose level was lower than 100 mg/dL or 5,55 mol/L [15, 17, 18]. Only in MS group the median was greater than 100 mg/dL, indicating hyperglycemia according to the inclusion criteria described by Tvarijonaviciute et al. [7]. More studies are needed to define the cutoff point for the classification of hypoglycemic or normoglycemic animals [19], as well as the blood glucose cutoff point to cause insulin resistance. However, in this study the serum insulin and insulin resistance has not been evaluated. The comparison of induced obesity studies and spontaneous obesity studies can be misleading, because in spontaneous obesity it is not possible to accurately determine the time of fat mass accumulation and it can determine the development of insulin resistance.

The high SBP was the less common of the criteria among the four groups. However, the median values of the four groups were within normal values, which are values at low risk of developing lesions in target organs such as kidneys, retina, heart, and brain, worsening morbidity and mortality [20, 21]. Similar results were observed in 19 dogs with BCS 7–9/9 and 19 animals with BCS 5/9 [20].

Dogs can develop many of the components of the MSN: obesity [3, 4, 7, 17, 22, 23], insulin resistance [24], increased blood pressure [20, 24, 25], and hyperlipidemia [7, 13], and these changes were observed in 22% of the animals studied and in nearly 50% of dogs with BCS 8-9, with statistically significant difference from the other groups of this study.

In 22,87% dogs two or more inclusion criteria to MS group were observed, similar to another study that found 20% of 35 dogs with MS criteria [7]. Among the 69 dogs with BCS 8-9/9, 49,28% (34) had two or more inclusion criteria to MS group, while only 27,72% (28 of 101) of overweight dogs had it. Apparently the degree and duration of fatness and visceral/central adiposity have contributed to the dogs being classified in MS. However, further research is needed to develop a method of measuring central obesity that may contribute to the understanding of the metabolic changes of obesity.

Of the 62 dogs the fasting blood glucose was higher than 100 mg/dL in 39 patients (62,9%), hypertriglyceridemia was present in 57 (91,94%), hypercholesterolemia in 51 (82,26%), and SBP in 24 (38,71%) of dogs. However, Tvarijonaviciute et al. [7] observed elevated blood glucose in 31,43%, serum triglycerides in 8,57%, cholesterol in 14,43%, and SBP in 28,57% of dogs included in the MS group, differing from our findings. The reasons for these differences have not been determined, but the variation in collection time and the objectives of the studies (effects of weight loss versus epidemiological study) may have contributed to the results obtained. Hyperglycemia has been reported in previous studies, but the cutoff point is a factor that differs largely [13, 17–19, 26], but was not present in other studies [5, 27]. In 39 dogs that had blood glucose levels above 100 mg/dL, 13 had values between 120 and 159 mg/dL. According to criteria adopted in this study fasting hyperglycemia was observed.

Dogs from MS group were 2,91 times more likely to develop hyperglycemia when compared to dogs with BCS 4-5. It is demonstrated that the adiposity increases the risk of elevated blood glucose or hyperglycemia, as previous studies [12, 13, 17, 18, 26, 27].

The occurrence of spontaneous hypercholesterolemia, hypertriglyceridemia, and obesity [2, 7, 14, 15, 28] as the induced obesity [3] has been described. Our results differ from these studies because, between the dogs with inclusion criteria for MS, 20 had moderate hypercholesterolemia and eight severe hypercholesterolemia. However, most obese dogs presented cholesterol values below (>750 mg/dL) the risk limits for the development of atherogenic disease [4, 16, 29] while 12,95% of 69 dogs had cholesterol in the risk range for the development of atherosclerosis. In 19 dogs for MS group only two dogs had severe hypertriglyceridemia, as described in obese dogs [13, 18, 26, 29]. There are rare reports of obese dogs with values above 1000 mg/dL and in this study, only two dogs had increased values.

Occurrence of hypercholesterolemia was like other studies [7, 12, 18, 28, 29]. The hypertriglyceridemia (43,48%) was present in dogs with BCS 8-9, higher than observed for Brunetto et al. [29]. However, it was not possible to assess whether this is due to the inclusion in the MS group or the influence of adiposity, but the risk of developing hypertriglyceridemia was lower than 10 times in obese animals than in those with normal weight and overweight.

The frequency of hypertension was higher in groups with score 8-9 and MS (27,54% and 38,71%, resp.) without difference when compared. This value is higher than that described by other authors [20, 24]. Rocchini et al. [30] found that weight gain caused an increase in SBP, while other researchers found no correlation between obesity and SBP [25]. Contributing to the claims of the latter authors, a recent study found that 10 of 35 dogs had SBP higher than 160 mmHg and with the weight loss eight dogs remained with the SBP at the same levels [10]. The differences could be due to population differences, breed, age, and time of adiposity in dogs, as well as the absence of comorbidities in this group. Also, obesity may be a risk factor in the development of hypertension. When comparing the dogs of BCS 8-9 and MS group with others, the chance of developing hypertension decreases in 8 : 33 times (odds ratio) in the BCS 4-5 and BCS 6-7 groups. The fact that the dogs with MS brings more risks in the development of hypertension may be important in obese dogs with other comorbidities that predispose to hypertension, as endocrine and kidney diseases. This fact justifies the importance of adopting a metabolic classification as proposed by Tvarijonaviciute et al. [7]. It is important to conduct further studies in dogs with comorbidities and to define inclusion criteria for MS.

In obese dogs, hypertension can occur by different mechanisms including the activation of the renin-angiotensin-aldosterone axis [31], hyperadrenergic activation and insulin resistance causing hyperadrenergic activation [32], and increased production of angiotensin and proinflammatory cytokines [17, 31, 33]. The moderate to severe increase associated with other changes in the MS group such as dyslipidemia and elevated blood glucose, and probably resistance insulin, may contribute to the development of complications associated with hypertension including stroke (CVA), retinopathy, choroidopathy, ventricular hypertrophy, albuminuria/microalbuminuria, and nephropathy [12, 17].

Previous studies have found a few animals with severe metabolic parameters [10, 14, 18]. It was not possible in this study to indicate the causes of these differences, but the fatness can contribute to higher levels of metabolic parameters. The highest number of animals studied and less time collecting samples compared to that performed may be one of the causes of these differences, which pointed out that these authors aimed to compare the MS parameters before and after loss weight.

Schnauzer dogs may have primary dyslipidemia. Therefore, the inclusion of this breed in this study on the prevalence of MS may be debatable. However, the authors chose to include them, since all the criteria for MS were met, so the dogs of this breed had to have hypertension, blood glucose >100 mg/dL, hypertriglyceridemia, and hypercholesterolemia. The five dogs of this breed in the study had all the criteria of MS but have a previous diagnosis of* diabetes mellitus* and moreover were aged between 3 and 8 years.

In MS group the probability of a dog being neutered was 79,03%, with 4,09 likely (odds ratio) to have neutered dogs with MS compared to neutered dogs with scores 4-5 and 6-7. This result is similar to those described, because castration increases the risk of obesity and probably also increases the risk of the development of the factors leading MS [18].

Age at MS group showed the same behavior described earlier in obese dogs; in other words, it was more common in older dogs (over 6 years of age). Apparently, age seems to influence the development of alterations compatible with MS, probably due to longer adiposity.

Some problems were detected in this study, such as the inability to determine the risk factors for the inclusion of the 62 dogs in the MS group because when it held the division into categories by breed, age, sex, neutered or not spayed, type of feed, or level of physical activity each category had a small number of animals. Another problem was the nonquantification of fat by dual-energy X-ray absorptiometry (DEXA) and its influence on severe increases in SBP, serum concentrations of cholesterol and triglycerides in the MS group dogs, and determining the time of adiposity of animals included in the study. The absence of serum insulin and HOMA test to identify impaired glucose tolerance and/or insulin resistance is another deficiency in this study. However, it is well established that animals overweight and obese may have hyperinsulinemia [5, 13, 18, 26, 27].

According to the criteria defined it was determined that significant portion (about 22%) of overweight and obese dogs have metabolic abnormalities consistent with MS. However, a longitudinal study of this population makes it possible to detect whether these animals are at risk of developing complications such as* diabetes mellitus*, atherosclerosis, heart attack, and stroke [25], which are described as complications of MS in humans, but the occurrence is questionable in dogs [19].

Based on the results we can say that the long-term changes may bring clinical changes. Therefore, we disagree with other authors who claim that this classification for the MS is unnecessary. There are currently not well-defined values cut to the serum concentration of triglycerides, cholesterol, and glucose levels as we have for the SBP, but it is important to try to set them on the basis of normal values. Future studies are needed to identify why some dogs develop metabolic changes (hyperglycemia, insulin resistance, hyperlipidemia, and hypertension) associated with obesity and others do not. The reasons dogs apparently do not develop the consequences of MS from other species like type II diabetes, atherosclerosis, and stroke also require confirmation as they are currently not known.

## 5. Conclusions

It follows that the metabolic syndrome according to the parameters defined in the literature for inclusion was observed in 22,87% of the animals studied and in 36% of dogs overweight or obese. Furthermore, the MS was most common in obese (49%) compared to overweight dogs (27%).

## Figures and Tables

**Table 1:** Median, minimum, and maximum values and number of observations (*N*) in 271 dogs grouped according to the BCS and inclusion criteria for metabolic syndrome.

		BCS 4-5	BCS 6-7	BCS 8-9	MS
		*N* = 101	*N* = 101	*N* = 69	*N* = 62
Cholesterol (mg/dL)	Average	196^a^	229^ab^	276^b^	418^c^
	Min–max	93–827	113–1055	131–827	175–1055
Triglycerides (mg/dL)	Average	124^a^	148^ab^	175.5^b^	299^c^
	Min–max	44–935	54–1005	74–1314	93–1314
Glucose (mg/dL)	Average	91^a^	94^ab^	97^bc^	108.5^c^
	Min–max	65–156	76–159	80–133	78–159
SH (mm/Hg)	Average	131^a^	135^a^	142^b^	144^b^
	Min–max	120–181	93–223	114–256	114–256
Age (years old)	Average	6^a^	8^b^	9^b^	8^b^
	Min–max	1–15	1–14	3–14	3–13

Medians followed by different letters in the same line showed differences (*p* < 0.05) according to the Kruskal-Wallis test. BCS: body condition scale and MS: metabolic syndrome.

**Table 2:** Spearman rank order correlations pairwise matrix from 62 dogs with two or more inclusion criteria for MS.

	Glucose	TG	COL	SBP	BCS	Age
Glucose		0.392171^*∗*^	0.078798	0.034454	0.041343	−0.170240
TG	0.392171^*∗*^		0.137775	−0.006817	0.072856	−0.198283
COL	0.078798	0.137775		−0.181512	0.016145	0.313763^*∗*^
SBP	0.034454	−0.006817	−0.181512		0.385798^*∗*^	−0.076325
BCS	0.041343	0.072856	0.016145	0.385798^*∗*^		0.285910^*∗*^
Age	−0.170240	−0.198283	0.313763^*∗*^	−0.076325	0.285910^*∗*^	

^*∗*^Marked correlations are significant at *p* < 0.05. TG: triglycerides, COL: cholesterol, SBP: systolic blood pressure, MS: metabolic syndrome, and BCS: body condition score.

**Table 3:** Absolute frequency and relative hyperglycemia (>100 mg/dL), hypercholesterolemia (>300 mg/dL), hypertriglyceridemia (>200 mg/dL), hypertension (>160 mmHg), castration, and sex in 271 dogs grouped according to body condition and inclusion criteria for MS.

		BCS 4-5	BCS 6-7	BCS 8-9	MS
Hyperglycemia	Yes/no	15/86^a^	28/73^b^	25/44^b^	23/39^b^
	%	14.85	27.72	36.23	37.09
Hypercholesterolemia	Yes/no	18/83^a^	29/72^abc^	29/40^b^	49/13^d^
	%	17.82	28.71	42.03	79.03
Hypertriglyceridemia	Yes/no	16/85^a^	28/73^b^	30/39^c^	57/5^d^
	%	15.84	27.72	43.48	91.94
SH	Yes/no	1/100^a^	13/88^b^	19/50^c^	24/38^c^
	%	0.09	12.87	27.54	38.71
Spayed	Yes/no	40/61^a^	57/44^bc^	49/20^cd^	49/13^d^
	%	39.61	56.44	71.01	79.03
Sex	F/M	57/44^a^	66/35^a^	46/26^a^	38/24^a^
	%	56.44	65.35	62.32	61.29

Followed proportions of different letters in the same line differed in the Chi-square test (*p* < 0.05). SH: systolic hypertension, MS: metabolic syndrome, and BCS: body condition score.
